# Altermagnetic Metal–Organic Frameworks

**DOI:** 10.1021/jacs.6c06455

**Published:** 2026-06-23

**Authors:** Diego López-Alcalá, Andrei Shumilin, José J. Baldoví

**Affiliations:** Instituto de Ciencia Molecular, 16781 Universitat de València, Catedrático José Beltrán 2, 46980 Paterna, Spain

## Abstract

Altermagnetism has recently emerged as a new class of spin compensated magnetic materials that exhibit momentum dependent spin splitting despite having zero net magnetization. The origin of these electronic signatures lies in symmetry operations that connect opposite spin sublattices while allowing spin splitting in momentum space. While most candidate materials identified so far belong to inorganic crystals with fixed lattice symmetries, the realization of altermagnetism ultimately requires platforms in which magnetic symmetry can be deliberately engineered. In this Perspective, we discuss how metal–organic frameworks (MOFs) provide a unique chemical platform to address this challenge. We first place altermagnetism in the broader context of magnetic and electronically active metal–organic networks, highlighting how reticular chemistry enables precise control over lattice geometry, dimensionality and electronic structure. We then discuss how these features position framework materials as promising candidates for realizing altermagnetism and highlight the key challenges that must be addressed to translate theoretical proposals into experimentally accessible systems. Finally, we outline emerging directions for realizing and controlling altermagnetism in coordination framework materials, which emerge as a versatile and powerful platform for exploring new paradigms in spintronics.

## Graphical abstract

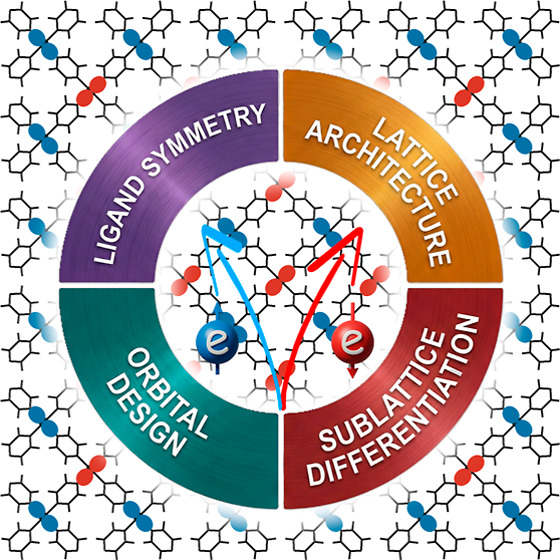

## Introduction

### Why Does Altermagnetism Need Chemistry?

Altermagnetism has been recognized as the scientific breakthrough in Physics 2024 and represents a fundamentally new form of magnetic order, redefining how spin polarization can arise in crystalline solids without net magnetization.^1–4^ By demonstrating that symmetry alone can generate momentum dependent spin splitting, altermagnetism bridges key concepts from ferromagnetism, antiferromagnetism and spin–orbit physics, offering an attractive route toward spin transport and spintronic functionalities without macroscopic magnetic fields.^5^ To date, however, the exploration of altermagnetism has been largely confined to a relatively narrow set of inorganic materials.^6^

This raises a central question: if altermagnetism is governed by symmetry, where can symmetry itself be deliberately designed rather than merely identified? Dense inorganic crystals offer limited freedom in this regard, as their lattice topology, electronic structure and magnetic symmetry are tightly constrained by atomic packing. In contrast, chemistry and coordination chemistry in particular, provides a fundamentally different paradigm. Through the modular combination of metal centers and organic linkers, chemical synthesis enables the deliberate construction of lattices, sublattices and magnetic motifs with programmable geometry, connectivity and electronic character.^7,8^

In this Perspective, we explore the potential of metal–organic frameworks (MOFs)–recognized with the Nobel Prize in Chemistry in 2025– ^9,10^ to provide ideal and largely untapped platforms for altermagnetism. Rather than searching for altermagnetism within a fixed materials landscape, coordination frameworks allow symmetry to be engineered by design, thus transforming altermagnetism from a rare emergent phenomenon into a chemically accessible magnetic state. We critically examine the current theoretical and experimental challenges that must be overcome to realize altermagnetism in MOFs and outline emerging directions that exploit their unique structural and electronic versatility. Therefore, this Perspective aims to clarify why altermagnetism needs chemistry and how chemical design may ultimately redefine the scope, robustness and functionality of altermagnetic (AM) materials.

## Altermagnetism as a Symmetry-Driven Phenomenon

Altermagnets form a distinct class of collinear magnetic materials with two magnetic sublattices, alongside ferrimagnetic (FiM) and antiferromagnetic (AFM) materials. These three classes are distinguished purely by symmetry considerations, specifically by the presence and character of symmetries that relate the two sublattices.^1^ In the absence of any sublattice-relating symmetry, a material is classified as FiM, whose macroscopic properties closely resemble those of ferromagnets. A material is identified as AFM when such a symmetry exists and, upon acting on the electron momentum, either preserves *it* (e.g., a translation *t*) or reverses *it* (e.g., inversion *i* or the combined *it* operation). The third possibility defines the AM phase, where sublattice-relating symmetries exist but act on electron momentum in a more complex manner. Altermagnets combine hallmark properties of both antiferromagnets and ferromagnets.^6^ Like AFM materials, they exhibit zero net magnetization, rendering them robust against external magnetic fields and feature linear magnon dispersion. At the same time, similar to ferromagnets, their spin-split electronic bands enable electrical detection of magnetic order parameters via the anomalous Hall effect^11,12^ and tunneling magnetoresistance.^13^ Moreover, spin injection into adjacent materials is possible due to spin-polarized currents arising from the spin splitting effect,^14^ which is a strong analogue of the much weaker spin Hall effect driven by spin–orbit coupling (SOC).^15^

Usually, AM materials are further classified into *d*-, *g*- and *i*-wave symmetry classes, characterized by two, four and six planes, respectively, in the spin splitting of the electronic dispersion. While all altermagnets share zero net magnetization and nonrelativistic band splitting, their functional responses differ markedly. Notably, only *d*-wave altermagnets support spin splitting currents in the linear (Ohmic) response regime, whereas in the other symmetry classes analogous effects emerge only as higher-order responses to an applied electric field.^16^

Because altermagnets are defined by nonrelativistic electronic properties, it is conventional to describe AM materials using nonrelativistic symmetry notation. In this description, each relevant symmetry operation is written as a pair [*E* | *g*_0_] or [*C*_2_ | *g*_sf_]. Here, the first symbol denotes the operation in spin space, where *E* represents spin conservation and *C*_2_ a 180° spin rotation (spin flip), while the second symbol specifies the corresponding operation acting on the crystal lattice. The coexistence of these two types of symmetry operations —[*E* | *g*_0_] and [*C*_2_ | *g*_sf_]—is what defines an AM state itself and determines its specific symmetry class. To illustrate this idea, Figure 1a shows a simple two-dimensional (2D) model of an altermagnet composed of two magnetic sublattices.^17^ Each sublattice is formed by a set of orbitals related by a 90° rotation and occupied by electrons with opposite spin orientations. In this example, the [*C*_2_ | *g*_sf_] operation corresponds to a 90° rotation about the *z*-axis (*C*_4_), which maps one sublattice onto the other while reversing the spin. By contrast, two mirror planes, σ_1_ and σ_2_, act as [*E* | *g*_0_] symmetries, preserving the spin orientation.

**Figure 1:**
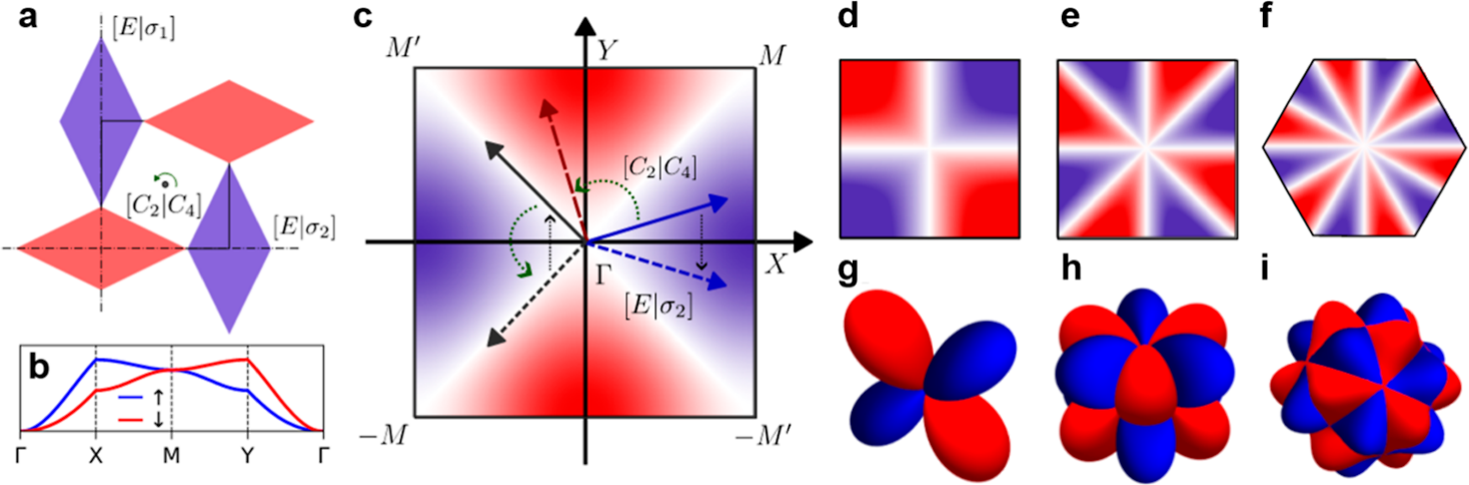
(a) Schematic illustration of a toy model of AM material belonging to the *d*-wave class. (b) Representative electron dispersion in such a material. (c) Diagram illustrating the action of symmetry operations on the electron dispersion: the [*C*_2_ | *C*_4_] operation preserves the band structure with a spin-flip, indicated by the red dashed arrow; the [E | σ_2_] operation preserves the band structure without a spin-flip, indicated by the blue dashed arrow. The consecutive application of these two symmetry operations to a wavevector along the Γ*M*’ direction preserves momentum while including a spin-flip, thereby preventing electron band splitting along this direction. (d–f) Representative band-splitting patterns in 2D *d*-, *g*- and *i*-wave altermagnets. (g–i) Representative angular dependences of the band splitting in 3D *d*-, *g*- and *i*-wave altermagnets.

Figure 1b shows the resulting electronic band structure. Owing to the AM symmetry, the bands exhibit a wavevector-dependent spin splitting that vanishes along specific high-symmetry directions. In three-dimensional (3D) systems, these directions generalize into nodal planes. Such spin splitting patterns are often summarized using schematic diagrams, as shown in Figure 1c.^18^ In these diagrams, regions of the First Brillouin zone with opposite sign of spin splitting are indicated by different colors. Applying a [*C*_2_ | *g*_sf_] operation to a given wavevector maps the electronic state onto one with the same energy but opposite spin, corresponding to a transition between regions of opposite color (red dashed arrow in Figure 1c). In contrast, a [*E* | *g*_0_] operation preserves both the energy and the spin, leading to a transition within regions of the same color (blue dashed arrow). Finally opposite wavevectors always belong to the same color region in the schematic representation. Along certain high-symmetry directions, specific symmetry operations that include a spin flip can map a wavevector onto itself, giving rise to nodal directions and planes. This is illustrated in Figure 1c, where the black arrow denotes a wavevector along the Γ*M*’ direction. In this case, the wavevector remains invariant under the successive application of the [*C*_2_ | *C*_4_] and [*E* | σ_2_] symmetry operations.

The wide variety of possible material symmetries gives rise to distinct angular dependences of spin splitting in altermagnets. In Figure 1d–f, we present schematic diagrams of the most common spin splitting patterns in the first Brillouin zone of 2D *d*-, *g*- and *i*-wave altermagnets. Figure 1g–i shows the typical angular dependences of the electron-band spin splitting in 3D *d*-, *g*- and *i*-wave altermagnets.

## Magnetic MOFs as Symmetry-Engineered Quantum Materials

### General Background of Magnetic MOFs for Spintronics

Over the past decade, coordination frameworks have evolved into chemically programmable platforms for controlling electronic and magnetic degrees of freedom.^19–23^ Their modular nature enables direct coupling between ligand symmetry and coordination geometry, providing a level of structural and electronic tunability that is difficult to achieve in conventional solids. Within this context, magnetic MOFs have emerged as versatile systems for engineering spin-dependent functionalities through chemical design, with organic ligands mediating exchange interactions that stabilize cooperative magnetic behavior despite the low density of spin centers.^24–28^

Early studies established the chemical principles required to achieve simultaneous magnetism and electronic transport in MOFs. In particular, redox-active quinoid ligands enabled long-range magnetic order high Curie temperatures together with measurable conductivity, while π–d conjugated frameworks provided an alternative route to delocalized magnetism and semiconducting transport (Figure 2a).^29–31^ These works identified metal–radical exchange and π–d hybridization as key mechanisms to overcome the intrinsically weak magnetic interactions of coordination frameworks.^32^

**Figure 2:**
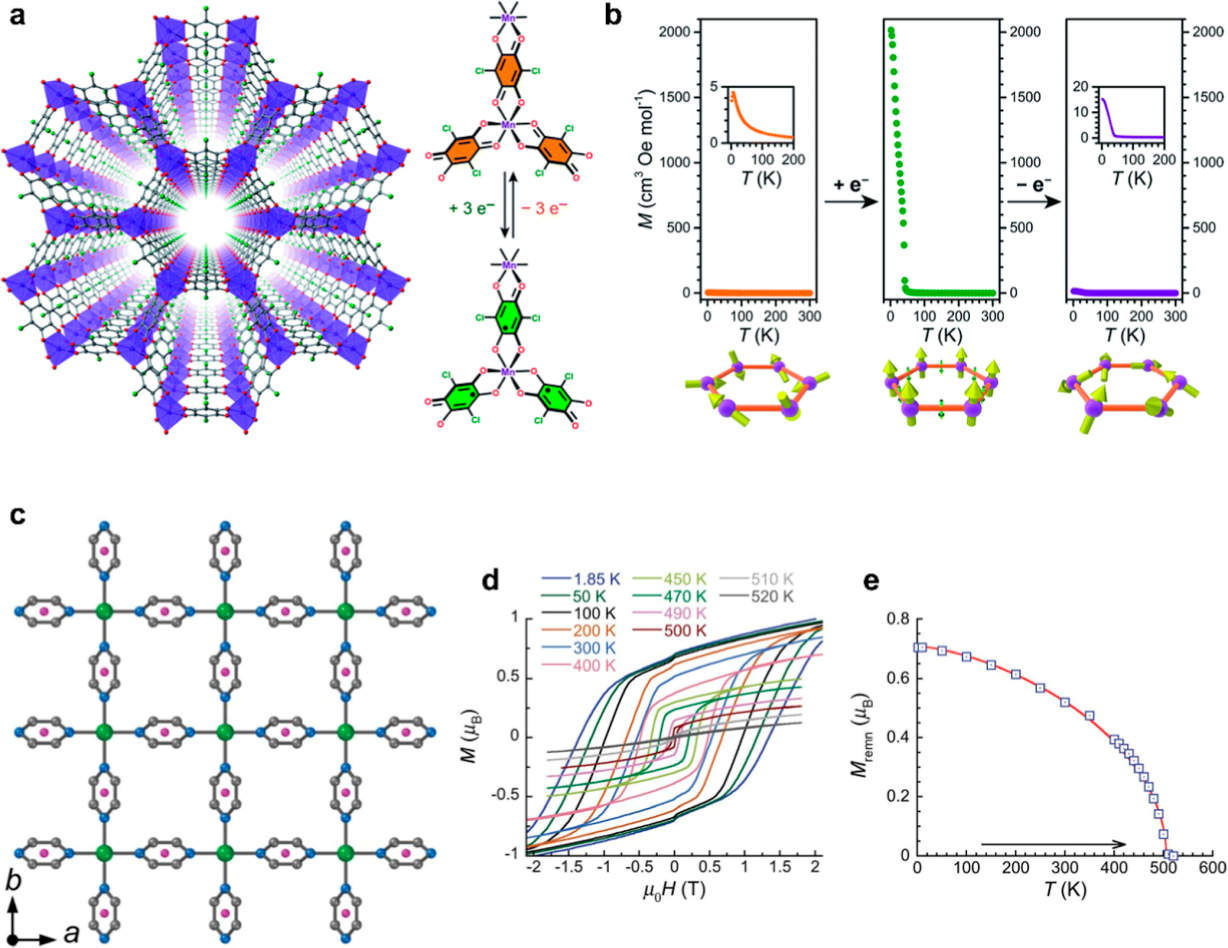
(a) Crystal structure of (Et_4_N)_2_[Mn_2_L_3_]·*x*DMF, as viewed along the crystallographic *c* axis. Violet octahedra represent Mn atoms; green, red, and gray spheres represent Cl, O, and C atoms, respectively. Adapted from ref 31. Under the terms of the CC-BY 3.0 license. Copyright 2019 Royal Society of Chemistry. (b) Variable-temperature field-cooled magnetization data for collected under an applied dc field of 10 Oe in (Et_4_N)_2_[Mn_2_L_3_]·*x*DMF. (c) Crystal structure of Li_0.7_[Cr­(pyz)_2_]­Cl_0.7_ 0.25­(THF) viewed along the *c* direction. (d) Magnetization versus applied dc magnetic field data and (e) temperature dependence of the remnant magnetization in Li_0.7_[Cr­(pyz)_2_]­Cl_0.7_ 0.25­(THF), from 1.85 to 520 K. Adapted with permission from ref 42. Copyright 2020 American Association for the Advancement of Science.

More recently, a new generation of magnetic MOFs has demonstrated that long-range magnetic order can persist in reduced dimensionality and atomically defined architectures. Layered van der Waals (vdW) frameworks retain magnetic order down to few-layer thickness, while on-surface synthesized 2D networks exhibit cooperative magnetism with sizable coercivity and well-defined anisotropy.^33–36^ At the same time, chemically complex lattices incorporating mixed valence and spin centers have enabled the realization of intertwined magnetic substructures and competing exchange interactions within a single framework.^37–39^

Among the different chemical strategies, systems based on redox-active ligands have consistently achieved the highest magnetic energy scales.^26^ Radical-mediated exchange interactions in coordination networks have enabled magnetic ordering temperatures approaching those of inorganic materials, including record values up to ∼400 K in V­[TCNE]_*x*_ systems and above 500 K in modified Cr–pyrazine frameworks, together with sizable coercivities and electronic conductivity (Figure 2b).^40–43^ These results highlight the central role of metal–radical coupling in enhancing magnetic robustness and positioning coordination frameworks as viable platforms for spintronic applications.

At the same time, coordination frameworks naturally accommodate multiple inequivalent magnetic sites, mixed-valence configurations and competing exchange pathways within a single lattice. This chemically encoded complexity enables the realization of magnetic states that go beyond conventional ferromagnetic (FM), FiM and AFM order, while offering a level of design flexibility that remains largely inaccessible in dense inorganic crystals.

### Framework Chemistry as a Symmetry Engine

A defining feature of MOFs is their exceptional versatility in constructing and controlling symmetry. In coordination frameworks, symmetry is not imposed by dense atomic packing, but deliberately constructed through the choice of metal nodes, organic linkers and their modes of connectivity. As a result, MOFs and related coordination networks provide access to an unusually rich symmetry landscape, encompassing a broad range of point groups, lattice symmetries and dimensionalities within a chemically unified materials platform.^44–47^ Notably, while most MOFs adopt 3D architectures, a large fraction of experimentally realized magnetic frameworks are based on 2D or layered structures, where reduced dimensionality facilitates the emergence and control of magnetic interactions. In addition, layered architectures naturally enable magnetic anisotropy, interlayer decoupling and external tunability through stacking or electric-field effects, making them particularly attractive platforms for low-dimensional magnetism and symmetry-driven electronic phenomena.^26^

This versatility originates from the modular nature of coordination chemistry. Organic linkers with well-defined geometry, rigidity and connectivity act as symmetry defining elements that propagate their local point symmetry into the extended lattice.^48^ By varying linker shape, connectivity and binding directionality, coordination frameworks can be rationally assembled into a wide range of lattice architectures, including square, rectangular, honeycomb, Kagome and more complex topologies (Figure 3).^49,50^ Periodic lattices such as square and honeycomb networks have enabled robust long-range magnetic order and Dirac-like electronic behavior in coordination frameworks,^51–53^ while frustrated geometries promote competing interactions and unconventional magnetic states associated with geometric frustration and quasiperiodic order.^54,55^ Importantly, Cairo pentagonal tiling, long associated with exotic magnetic ground states in inorganic materials, have recently been realized in MOFs through isoreticular design, demonstrating the synthetic accessibility of such unconventional symmetries within coordination chemistry (Figure 4a).^56^ Low-symmetry and mixed-topology lattices further expand this landscape by enabling multiple inequivalent magnetic sites and competing exchange pathways, giving rise to noncollinear spin configurations and frustration-driven behavior (Figure 4b).^57^ The modularity of coordination frameworks has also recently enabled the incorporation of heavier 4d transition metals, further expanding the accessible chemical and symmetry space of magnetic MOFs.^58^ Together, these results highlight how chemically encoded lattice architectures enable magnetic states that remain difficult to access in conventional inorganic crystals.^59^

**Figure 3:**
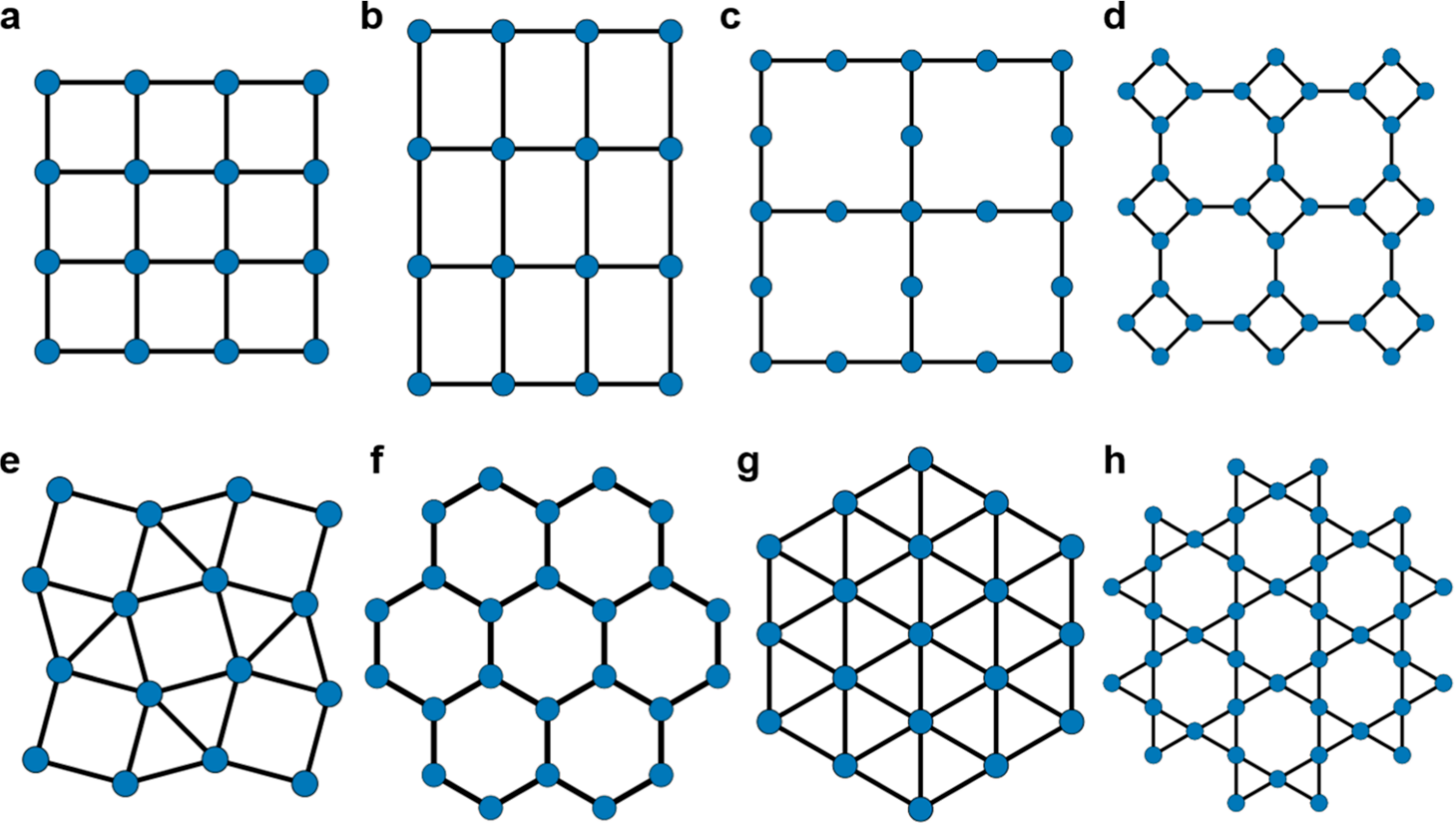
Representative 2D topologies hosting magnetic sublattices in layered MOFs. The diagrams illustrate the nodes (blue spheres) and linkers (black lines) corresponding to the RCSR codes and Schläfli symbols: (a) sql (4^4^), (b) rectangular distortion of the sql net, (c) Lieb lattice, (d) fes (4.8^2^), (e) tts (snub square, 3.3.4.3.4), (f) hcb (honeycomb, 6^3^), (g) hxl (3^6^) and (h) kgm (Kagome, 3.6.3.6). These geometries exhibit distinct symmetry-breaking features and sublattice interplays crucial for emerging AM phases.

**Figure 4:**
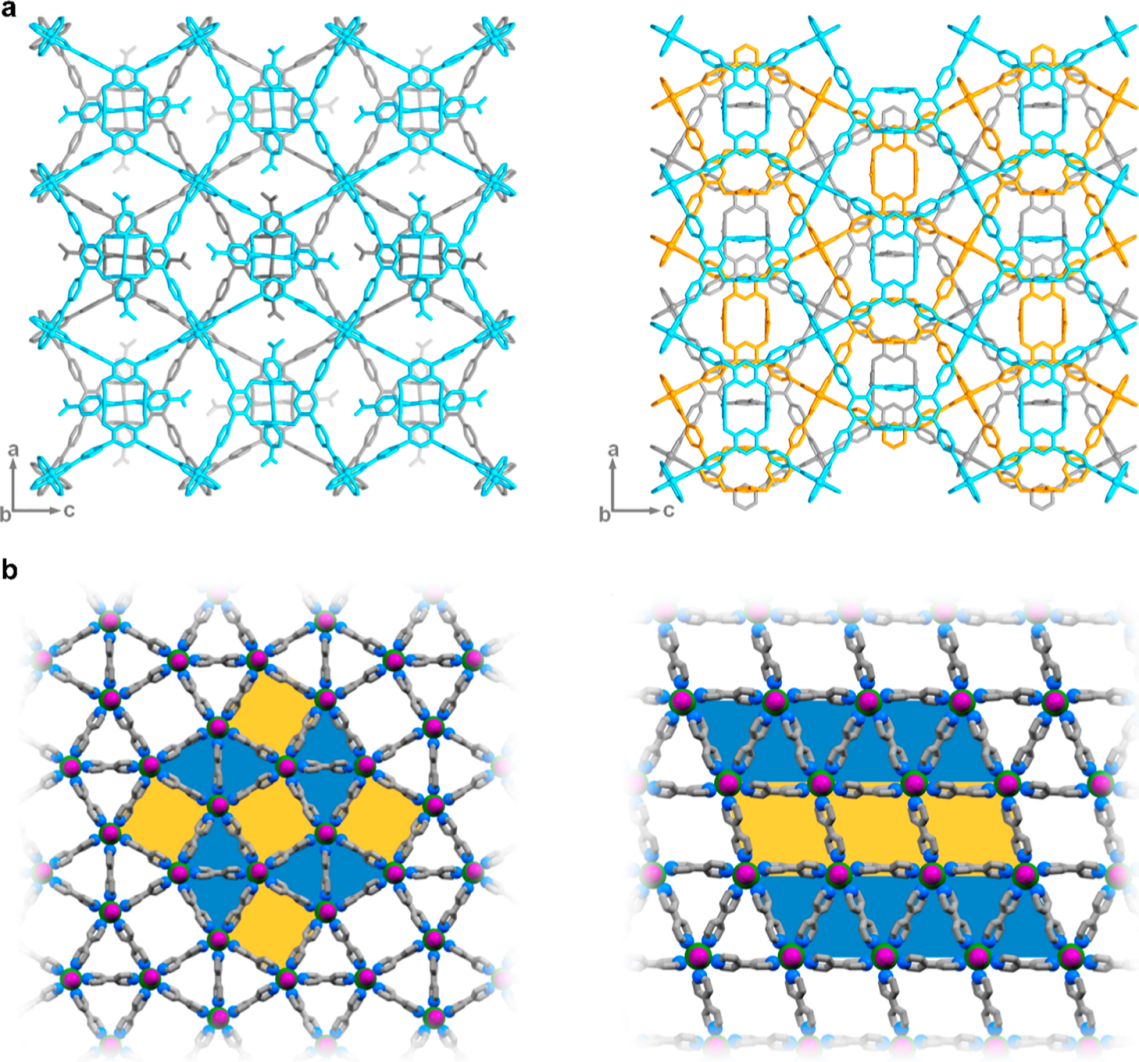
(a) Views of stacking layers in mcm-MOF-1­(left) and mcm-MOF-2 (right). Adapted with permission from ref 56. Copyright 2024 Elsevier. (b) Single-crystal X-ray structure of Gd (left). Single-crystal structure of the trace impurity phase Gd′ (right). Green, purple, blue and gray spheres represent Gd, I, N, C and H atoms, respectively. Adapted from ref 57. Copyright 2021 American Chemical Society.

Finally, the inherent softness and modularity of coordination frameworks allow symmetry to be modified well beyond the initial synthesis step. Experimental studies on layered magnetic coordination polymers and 2D MOFs have shown that presynthetic ligand functionalization, guest inclusion, redox chemistry and controlled postsynthetic transformations can systematically alter local coordination environments, interlayer registry and effective symmetry, while preserving crystallinity, magnetic order and the underlying network topology.^60^ This ability to generate isoreticular families of closely related frameworks, in which symmetry, electronic structure and magnetic interactions can be tuned in a controlled and chemically encoded manner, is essentially inaccessible in rigid inorganic crystals.^34^ In the context of altermagnetism, such chemical agility represents a unique opportunity to iteratively explore and refine symmetry conditions, transforming symmetry from a fixed structural constraint into a continuously adjustable design variable.

Taken together, these features position MOFs as a chemically programmable platform in which symmetry can be explored, tuned and deliberately engineered across an exceptionally broad parameter space. This intrinsic symmetry versatility provides the conceptual foundation for realizing unconventional magnetic states in framework materials and establishes altermagnetism as a chemically programmable property rather than a constraint imposed by existing crystal lattices.

## Emergence of Altermagnetism in MOFs

### Altermagnetism Meets Framework Materials

Initial theoretical efforts have appeared only recently, demonstrating the emerging potential of MOFs for altermagnetism. In a seminal contribution, Che et al. proposed the first realization of AM spin splitting in MOFs by engineering tetragonal lattices based on M­(pyz)_2_ networks, with M = Ca and Sr (Figure 5a).^61^ In these systems, an effective spin polarization of the ligand scaffold induces unconventional momentum-dependent spin splitting in the absence of net magnetization. This behavior originates from the symmetry-enforced coupling between magnetically compensated sublattices through the combined spatial and spin operation [*C*_2_ |*C*_4*z*_], which gives rise to a characteristic *d*-wave AM anisotropy.

**Figure 5:**
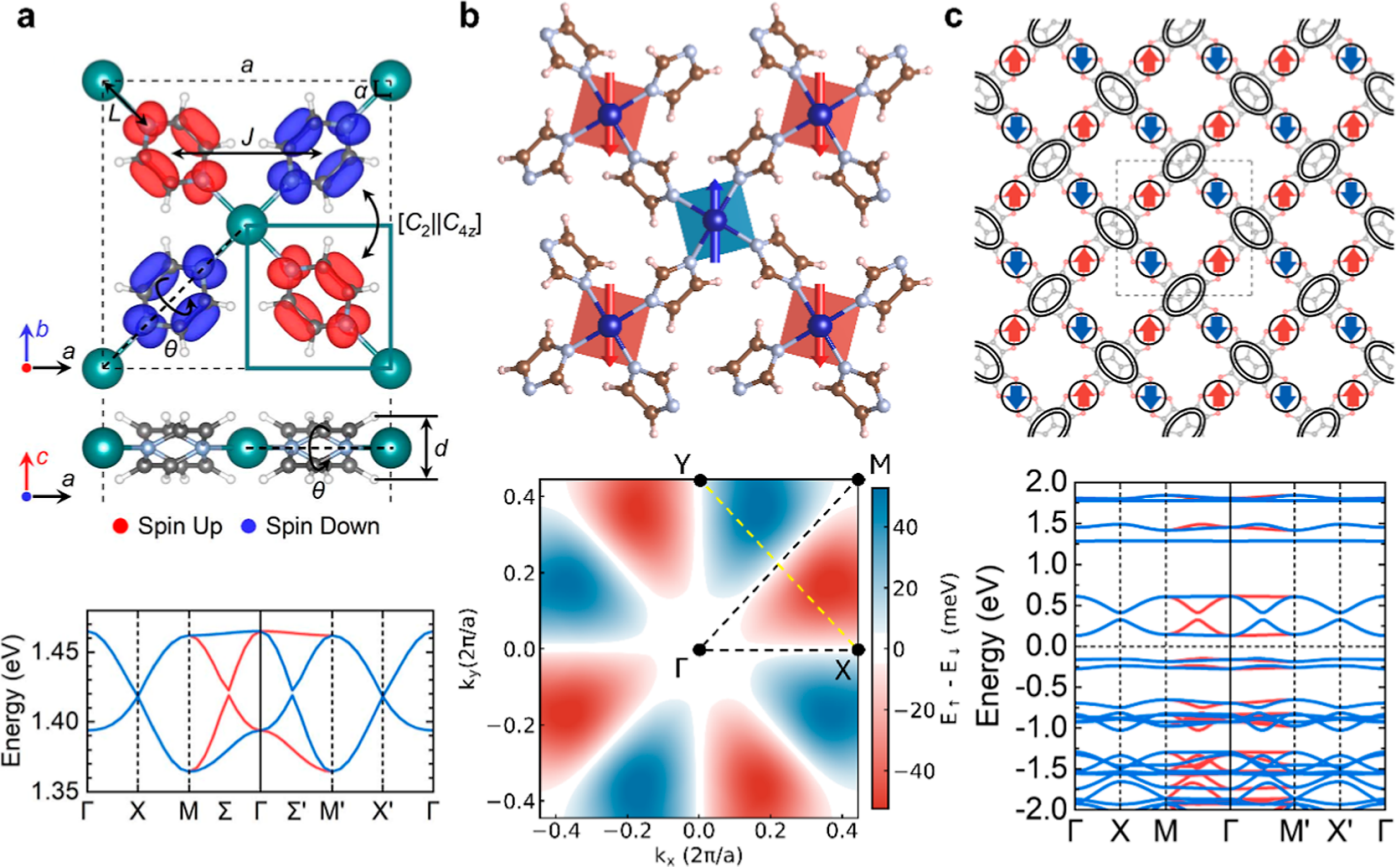
(a) AM 2D Ca­(pyz)_2_ and its electronic band structure. Green, gray, blue, and white balls represent Ca, C, N, and H atoms, respectively. Adapted from ref 61. Under the terms of the CC-BY-NC 3.0 license. Copyright 2024 Royal Society of Chemistry. (b) Representative imz-based 2D MOF antiferromagnetically coupled and its spin splitting in VBM along 2D MOF plane. Adapted from ref 62. Copyright 2026 American Chemical Society. (c) Assembly of *t*-Cr_2_[Pyc-O_8_] from Cr and Pyc-(OH)_8_ molecules and its electronic band structure. Blue, gray, and red balls represent Cr, C, and O atoms, respectively. Adapted with permission from ref 65. Copyright 2025 American Physical Society.

Building on initial lattice-specific realizations, subsequent works have established more general design principles for altermagnetism in coordination frameworks. In particular, ligand symmetry has been identified as a central chemical lever to control the spin space group of MOFs, enabling symmetry breaking between magnetic sublattices through the use of appropriately designed organic linkers (Figure 5b).^62^ This approach demonstrates that AM spin splitting can be directly encoded at the molecular level, with specific symmetry operations emerging from the ligand geometry and propagating throughout the lattice. Importantly, this strategy is not limited to a given topology but can be extended across a broad class of coordination networks. Representative examples include Cairo-type pentagonal 2D MOFs, where unconventional lattice symmetry enables AM spin splitting within geometrically frustrated architectures.^63^ Together, these results establish a general route toward symmetry-driven design of AM states in coordination frameworks.

Beyond lattice geometry, frontier molecular orbital engineering (FMOE) has emerged as a complementary mechanism to stabilize altermagnetism. By controlling the symmetry and energetic alignment of ligand-centered orbitals hybridized with metal *d* states, AM ground states can be achieved while preserving overall magnetic compensation, with predicted ordering temperatures approaching ∼180 K.^62,64^ This highlights that orbital design, rather than lattice symmetry alone, can dictate the emergence and robustness of AM phases.

In parallel, symmetry-first approaches have demonstrated that altermagnetism in MOFs can be designed a priori by mapping abstract symmetry criteria onto chemically realizable lattice architectures (Figure 5c).^65^ These methods identify families of coordination networks capable of hosting AM states based purely on their symmetry properties, providing a predictive framework that shifts the field from material discovery toward rational design. Building on this concept, recent works have shown that AM symmetry can be coupled to additional ferroic order parameters, enabling electric-field control of AM spin textures through ferroelectric switching in hybrid MOFs.^66–68^

While most proposals have focused on single-layer coordination networks, recent studies have extended altermagnetism to multilayer architectures, where interlayer symmetry operations provide an additional degree of freedom. In bilayer MOFs, spin-compensated sublattices can be separated across different layers and connected through combined symmetry operations, enabling external electric fields to modulate the AM electronic structure via layer polarization.^69^ More recently, these concepts have also been generalized to bulk 3D MOFs, where symmetry-enforced spin splitting can emerge in fully periodic frameworks, further expanding the design space beyond low-dimensional systems.^66,70^ These advances position altermagnetism in MOFs as a symmetry-driven and chemically designable magnetic state.

Importantly, many of the proposed AM MOF structures are based on coordination motifs and network topologies that are already well established in MOF chemistry, including pyz-bridged lattices, redox-active frameworks and layered coordination networks. This suggests that the realization of altermagnetism does not require the discovery of entirely new chemical platforms, but rather the targeted adaptation of existing framework families toward symmetry-controlled magnetic states.

### Building MOF Altermagnets: Chemical Design Strategies

Building on the design strategies outlined above, altermagnetism in coordination frameworks can be rationalized in terms of a small set of chemically controllable figures of merit. Rather than being tied to specific compounds, these parameters capture the ability of MOFs to realize the symmetry operations and spin space groups required for AM order. Most current theoretical proposals of AM frameworks can be understood within this compact design space, and notably, many of these design parameters correspond to synthetic handles already routinely exploited in coordination chemistry. In practice, symmetry, lattice architecture and electronic structure in MOFs are commonly tuned through well-established strategies such as linker functionalization, reticular design and redox control. Moreover, several coordination motifs recurrently proposed for AM MOFs, including pyrazine-bridged lattices, layered coordination networks and redox-active frameworks, are closely related to experimentally realized magnetic and conductive MOFs synthesized through established solvothermal, ionothermal and molten-ligand methodologies.^71,72^ In particular, pyrazine-bridged and radical-based magnetic frameworks, which appear in multiple theoretical AM proposals, have already been experimentally obtained under relatively accessible synthetic conditions.^41,42^ As a result, the realization of AM frameworks builds directly on experimentally established coordination chemistries, with the central challenge lying in achieving the specific symmetry conditions required to stabilize altermagnetic states. In this section, we identify four key parameters that govern the emergence and control of altermagnetism in framework materials: ligand symmetry, lattice architecture and dimensionality, magnetic sublattice differentiation and orbital design. Together, they define a chemically accessible map for designing AM coordination networks.

- Ligand symmetry
  Ligand symmetry provides the primary molecular origin of lattice symmetry in coordination frameworks. The local point symmetry, directionality and connectivity of an organic linker determine how coordination motifs propagate through space, thereby dictating the global symmetry of the extended network. As a result, variations in ligand geometry or substitution pattern can modify lattice symmetry elements without necessarily altering the overall topology. However, in practice, ligand symmetry and framework topology are often strongly coupled, and changes in linker structure may induce alternative network architectures. Strategies such as isoreticular design and postsynthetic modification can help preserve lattice connectivity while tuning local symmetry, although achieving independent control over symmetry and topology remains experimentally nontrivial. For example, the replacement of centrosymmetric linkers such as pyz by lower-symmetry heterocyclic ligands can reduce the symmetry of the metal–organic lattice and enable AM spin splitting.^62^ This direct translation of molecular symmetry into lattice symmetry is a distinctive feature of coordination chemistry, allowing chemically similar frameworks to exhibit distinct spin space groups and AM anisotropies solely as a consequence of linker symmetry.
- Lattice architecture and dimensionality
  While ligand symmetry defines the local building blocks, the global network architecture determines which symmetry classes are accessible at the lattice level. By deliberately designing the underlying topology, coordination frameworks can be constructed to satisfy the symmetry requirements associated with AM order. For instance, square, rectangular, honeycomb, or Cairo-type lattices each support distinct rotational and symmetry elements that can stabilize different AM anisotropies.^73–75^ Within this architectural freedom, dimensionality emerges as a powerful additional degree of control. 2D coordination networks naturally support planar rotational and glide symmetries that favor momentum dependent spin splitting, while multilayer and stacked architectures introduce interlayer symmetry operations that enable additional control channels, such as layer resolved spin textures. Fully 3D frameworks further expand the accessible design space by allowing nonsymmorphic symmetries and complex spin space group operations that are inaccessible in reduced dimensionality.^70^ In this context, changes in dimensionality are not merely geometric modifications, but constitute an active symmetry engineering strategy that qualitatively alters the nature and tunability of AM states.
- Magnetic sublattice differentiation
  A central structural requirement for altermagnetism is the presence of magnetically compensated yet crystallographically inequivalent spin sublattices. Unlike conventional antiferromagnets, where opposite spins occupy symmetry equivalent sites related by simple spatial operations, AM frameworks require antiparallel spins to reside on distinct crystallographic positions with different local environments.^76^ This controlled inequivalence prevents the enforcement of spin degeneracy while preserving global magnetic compensation. Coordination frameworks provide several chemical routes to achieve this condition. Structures containing multiple metal sites per unit cell, mixed coordination geometries, or the coexistence of metal centered and ligand centered spin carriers can place spins on distinct Wyckoff positions or on sites of reduced symmetry.^26^ Mixed valence frameworks, heterometallic lattices and networks with inequivalent coordination nodes offer particularly natural pathways to this type of spin differentiation.
- Orbital design and energy scales
  Beyond structural symmetry, orbital design governs the electronic energy scales that determine whether AM order becomes experimentally observable. Controlling the symmetry, spatial distribution and energetic alignment of ligand frontier orbitals, FMOE is a key tool to enable the deliberate reorganization of exchange pathways and spin delocalization across the lattice. This orbital control can induce qualitative changes in magnetic order, including the stabilization of AM ground states and the switching between distinct AM anisotropies within the same structural platform.^62,64^ In addition to selecting symmetry allowed states, orbital design sets the magnitude of exchange interactions and spin splitting. Ligand centered spin polarization, nonbonding molecular orbitals and redox active frameworks enhance magnetic coupling and promote extended spin delocalization, leading to higher magnetic ordering temperatures and larger electronic spin splittings. In this way, orbital engineering complements symmetry design by transforming altermagnetism from a symmetry allowed possibility into an energetically robust and potentially functional magnetic phase.

Taken together, these four figures of design parameters establish altermagnetism in coordination frameworks as a designable and programmable property rather than a material-specific curiosity (Figure 6). The modular nature of framework chemistry enables the construction of entire families of AM materials in which symmetry, sublattice structure and orbital character can be independently tuned. This Perspective shifts the search for altermagnets from empirical discovery toward rational design, opening a pathway to chemically engineered AM platforms with controllable functionality and relevance for future spintronic and quantum technologies.

**Figure 6:**
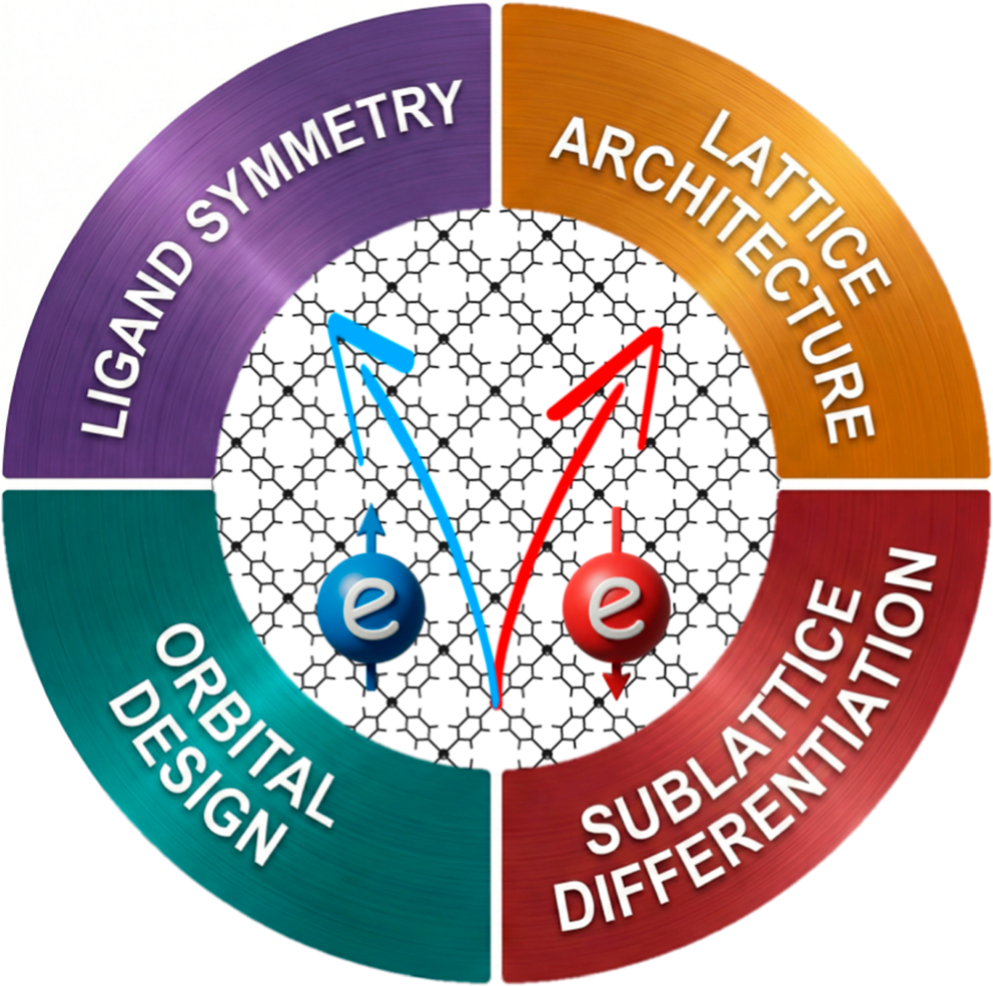
Schematic representation of discussed strategies for inducing altermagnetism in MOFs.

## Challenges, Prospects and Future Directions

### Fundamental Challenges

Despite the rapid conceptual and theoretical advances outlined above, the realization of altermagnetism in MOFs poses a distinct set of challenges that extend beyond conventional magnetic materials design. At the same time, it is important to highlight that most AM MOF candidates proposed to date remain at the theoretical level and are not yet accompanied by detailed synthetic protocols or experimental validation. This situation reflects the early stage nature of the field, where symmetry-based design has progressed faster than its experimental implementation. However, rather than indicating a lack of feasibility, this gap highlights the need to translate well-established synthetic strategies in MOF chemistry into symmetry-targeted approaches. In this sense, the realization of AM MOFs should be viewed not as the discovery of entirely new materials classes, but as the refinement of existing coordination frameworks toward specific symmetry and magnetic requirements. In this context, these challenges arise from the requirements that altermagnetism imposes on structural fidelity, experimental observables and energetic robustness. In the following, we critically assess the most relevant obstacles that must be addressed to translate symmetry-engineered AM concepts into experimentally accessible framework materials, while highlighting emerging experimental capabilities that make this goal increasingly realistic.

A central requirement underlying many of these challenges is the ability to access framework materials with well-defined structure and sufficient sample quality for advanced measurements. Encouragingly, several experimental advances directly address the challenges associated with identifying altermagnetism in coordination frameworks. The realization of electrically conductive MOFs as single crystals enables access to intrinsic, symmetry-resolved electronic and transport properties without averaging effects from grain boundaries, which is essential for detecting anisotropic and momentum-dependent responses.^77,78^ In parallel, the successful integration of MOFs into field-effect transistor (FET) architectures demonstrates their compatibility with device-level measurements, providing electrostatic control over carrier density and enabling gate-tunable transport signatures relevant to AM phases.^20,79^ Bottom-up fabrication strategies, including layer-by-layer and surface-assisted growth, further allow the preparation of oriented thin films with controlled thickness and crystallographic alignment, opening realistic routes toward angle-dependent transport and spectroscopic probes.^80^ Finally, the use of chemically programmable molecular building blocks permits the deliberate encoding of symmetry, orbital character and magnetic functionality at the molecular level, facilitating the rational construction of framework materials tailored for advanced magnetic and electronic characterization. Together, these developments indicate that the experimental tools required to probe altermagnetism in MOFs are rapidly converging toward practical implementation.

A further fundamental challenge lies in the experimental identification of altermagnetism itself. Because AM phases are fully compensated in real space, conventional magnetometry is inherently insufficient to distinguish them from collinear AFM order. Instead, altermagnetism must be identified through probes that resolve symmetry-dependent electronic structure either in momentum space, real space or through device-level responses and symmetry-sensitive optical probes (Figure 7). Momentum-resolved spectroscopies such as angle-resolved photoemission spectroscopy (ARPES) and its spin-resolved variant (s-ARPES) provide the most direct route to detect AM spin splitting by resolving band dispersions and spin textures in *k*-space.^81^ These approaches have been successfully applied to prototypical AM materials such as MnTe and CrSb, where symmetry-protected spin splitting has been directly observed.^82–85^ While these techniques require high-quality crystalline surfaces, they have also been applied to surface-supported 2D MOFs and coordination networks, demonstrating that momentum-resolved measurements are experimentally accessible in framework materials.^86,87^

**Figure 7:**
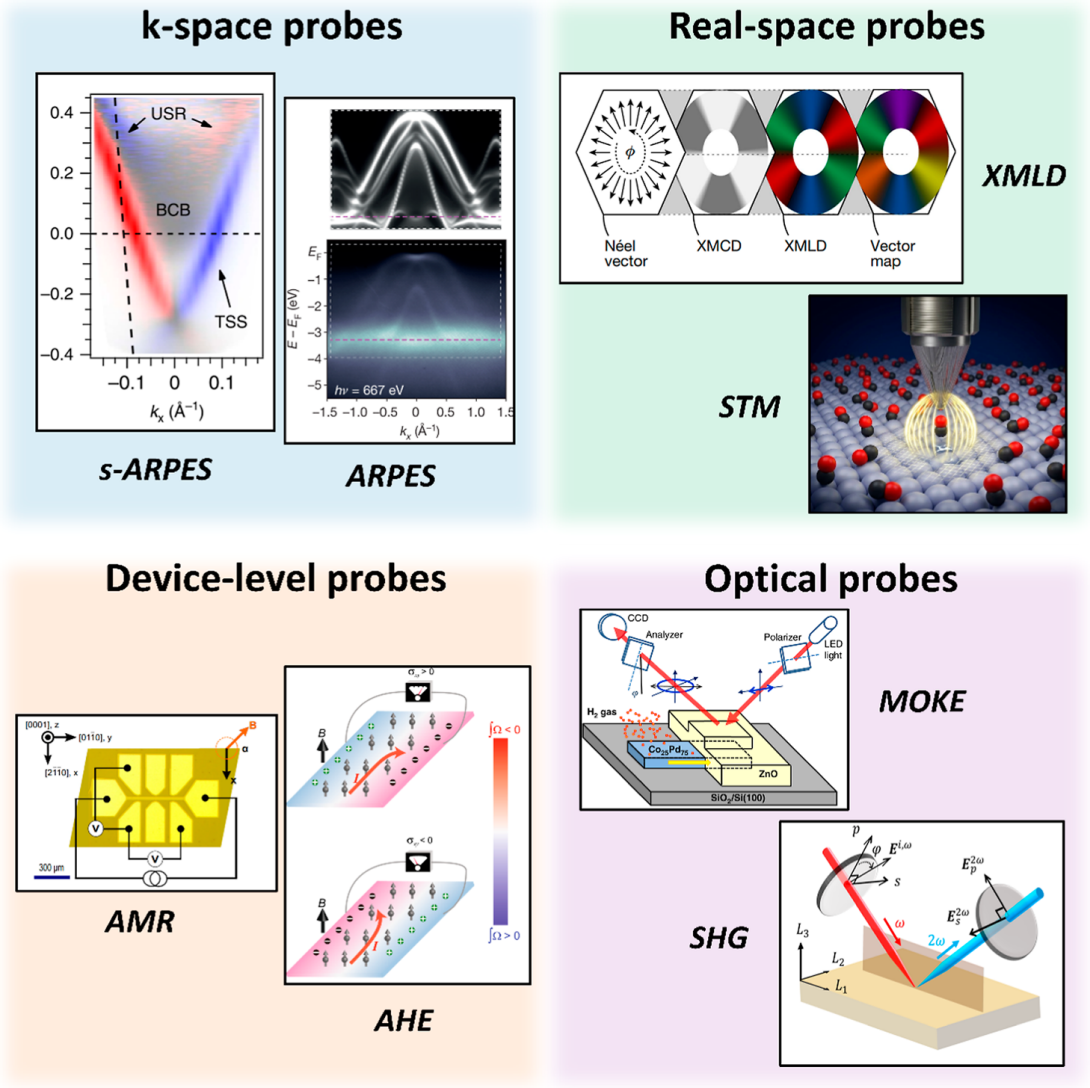
Schematic representation of different experimental techniques used for detecting altermagnetism. s-ARPES: Adapted from ref 81. Under the terms of the CC-BY 4.0 license. Copyright 2016 Springer Nature. ARPES: Adapted from ref 82. Under the terms of the CC-BY 4.0 license. Copyright 2024 Springer Nature. XMLD: Adapted from ref 90. Under the terms of the CC-BY 4.0 license. Copyright 2024 Springer Nature. MOKE: Adapted from ref 96. Under the terms of the CC-BY 4.0 license. Copyright 2019 Springer Nature. SHG: Adapted from ref 95. Under the terms of the CC-BY 4.0 license. Copyright 2022 Springer Nature. AMR: Adapted from ref 91. Under the terms of the CC-BY-NC 4.0 license. Copyright 2024 Springer Nature. AHE: Adapted from ref 93. Under the terms of the CC-BY 4.0 license. Copyright 2021 Springer Nature.

Real-space probes provide direct access to the microscopic symmetry breaking that defines AM order. Scanning tunneling microscopy (STM) can resolve local density-of-states modulations associated with spin-split electronic structures, enabling the visualization of intertwined charge and spin textures at the atomic scale.^88,89^ In parallel, synchrotron-based techniques such as X-ray magnetic circular dichroism (XMCD) and X-ray magnetic linear dichroism (XMLD), particularly when combined with photoemission electron microscopy (PEEM), allow real-space mapping of the magnetic order parameter. Recent experiments in MnTe demonstrate that the Néel-like AM vector can be reconstructed with nanoscale resolution by combining XMLD (sensitive to the spin-axis orientation) and XMCD (sensitive to time-reversal symmetry breaking), enabling direct imaging of domains, domain walls and topological textures such as vortices.^90^ These approaches go beyond spatially averaged probes and establish a framework for detecting altermagnetism in real space. Importantly, element-specific dichroic techniques such as XMCD have been already employed in MOFs to identify magnetic coupling and its microscopic origin, as demonstrated in 2D Cu-based MOFs exhibiting room-temperature ferromagnetism.^39^

From a device-oriented perspective, transport-oriented probes provide an experimentally accessible route to detect AM order through symmetry-dependent transport responses. In altermagnets, momentum-dependent spin splitting gives rise to characteristic anisotropic electronic transport even in the absence of net magnetization. As a result, anisotropic magnetoresistance (AMR) and related effects such as the anomalous Hall effect (AHE) constitute key signatures of altermagnetism,^91,92^ as they directly reflect the symmetry of the underlying spin-split electronic structure.^93^ These transport signatures have already been experimentally used to identify AM states in MnTe,^91^ establishing symmetry-resolved electronic transport as a robust and accessible fingerprint of altermagnetism. While direct measurements of these effects in MOFs remain limited, electrically conductive coordination frameworks have already demonstrated magnetotransport behavior and integration into device architectures.^19,20,77,78^ These advances establish a clear pathway toward symmetry-resolved transport measurements in framework materials, particularly in oriented thin films and device-compatible platforms, positioning device-level probes as a promising route for the experimental identification and functional exploitation of altermagnetism in MOFs.

Complementing momentum-, real-space and transport-based approaches, symmetry-sensitive optical probes provide a direct route to detect AM order through the electronic response of the system. Nonlinear optical techniques such as second harmonic generation (SHG) are intrinsically sensitive to broken inversion symmetry and have been widely employed in noncentrosymmetric and chiral coordination frameworks, where they probe symmetry-driven electronic and structural order.^94,95^ In parallel, symmetry-sensitive optical probes such as the magneto-optical Kerr effect (MOKE) directly access symmetry breaking in the electronic structure through the off-diagonal components of the optical conductivity tensor.^96^ Importantly, MOKE signals can arise in AM systems despite the absence of net magnetization, reflecting the underlying symmetry of the spin-split band structure.^97^ In hybrid MOFs and related materials, magneto-optical responses have already been shown to be electrically tunable and even switchable through the coupling between polarization and magnetic order, demonstrating the strong sensitivity of optical probes to symmetry-controlled spin textures.^98^ Together, these optical techniques provide a noninvasive and symmetry-resolved platform for identifying AM states in coordination frameworks.

Taken together, these results indicate that, although the experimental identification of altermagnetism in MOFs remains technically demanding, the experimental toolbox required to probe AM states is not fundamentally incompatible with coordination frameworks. Rather, its realization will likely emerge through the extension and integration of characterization methodologies that have already been independently demonstrated in magnetic and conductive MOFs. While altermagnetic MOFs remain, to date, a purely theoretical materials platform, the rapid convergence of symmetry-guided design principles, reticular synthesis strategies and advances in framework magnetism strongly suggests that the realization of experimentally accessible AM coordination networks is an achievable objective. In this context, the central challenge is no longer the conceptual possibility of altermagnetism in MOFs, but the targeted synthesis of frameworks simultaneously fulfilling the stringent symmetry, magnetic and electronic requirements needed to stabilize and experimentally resolve AM states.

A final fundamental challenge concerns the overall robustness of AM phases against thermal fluctuations, structural disorder, and environmental perturbations. As summarized in Table 1, theoretical proposals of AM MOFs predict momentum-dependent spin splittings typically in the range of 10–300 meV. While these values are already substantial and comparable to many 2D and 3D inorganic altermagnets, they remain significantly below the largest reported nonrelativistic AM spin splittings, which reach values close to ∼1 eV in inorganic systems such as CrSb.^85^ This gap highlights an important challenge for framework-based altermagnets, namely the need to further enhance electronic energy scales through stronger exchange interactions and increased spin delocalization. In parallel, a marked disparity persists in magnetic ordering temperatures. Whereas altermagnetism in inorganic materials has been identified in systems exhibiting magnetic order up to and including room temperature,^84^ predicted ordering temperatures for MOF-based altermagnets generally do not exceed ∼200 K. More broadly, within magnetic MOFs, transition temperatures approaching ambient conditions have only been achieved in frameworks incorporating redox-active ligands, where metal-radical exchange dominates,^42^ while systems relying primarily on metal–metal superexchange typically order at cryogenic temperatures. Establishing a practical viability of AM phases requires a thorough understanding of their behavior under realistic and nonideal conditions. Recent theoretical studies on inorganic lattices have demonstrated that the AM spin-splitter effect is surprisingly robust against structural imperfections.^99^ The momentum-dependent splitting remains resilient even when a high fraction of the lattice sites contain impurities with a scattering strength comparable to the nearest-neighbor hopping parameter. Beyond such intrinsic defects, external environmental perturbations also fundamentally influence the magnetic response. Rather than merely suppressing the effect, mechanical factors like epitaxial strain have been shown to actively stabilize and tune the AM phase in thin films such as RuO_2_.^100^ Therefore, given the inherent structural flexibility and typical defect profiles of MOFs, exploring how framework materials respond to both structural vacancies and environmental strain represent a critical frontier for future research and for the development of functional devices based on AM MOFs.

**Table 1:** Structure Type, AM Anisotropy, Spin Splitting (*E*_S_, in meV), Néel Temperature (*T*_N_, in K) and Level of Theory Used in Reported AM MOFs in Bibliography

	structure type	AM anisotropy	*E*_s_	*T*_N_	method	ref.
Ca(pyz)_2_	monolayer	d-wave	-	15.5	HSE06	^61^
Sr(pyz)_2_	monolayer	d-wave	-	11.5	HSE06	^61^
Cr(DAind)_2_	monolayer	d-wave	84	25	HSE06	^62^
Cr(diz)_2_	monolayer	d-wave	16	112	HSE06	^64^
Cr(c-pyr)_2_	monolayer	d-wave	30	177	HSE06	^64^
Cr(f-pid)_2_	monolayer	d-wave	37	183	HSE06	^64^
t-Cr_2_(Pyc-O)_8_	monolayer	d-wave	184	57	PBE + U	^65^
Cr(imz)_2_	monolayer	g-wave	65	37	HSE06	^62^
Cr(DApent)_2_	monolayer	g-wave	30	58	HSE06	^62^
Ru_2_(TCNQ)_2_	monolayer	g-wave	100	24	PBE + U	^63^
Cr(tcb)_2_	bilayer	d-wave	162	205	HSE06	^69^
Cr(hcb)_2_	bilayer	d-wave	314	85	HSE06	^69^
[C(NH_2_)_3_]Cr(HCOO)_3_	bulk	d-wave	20	-	PBE + U	^66^
Co(pymo)_2_	bulk	g-wave	75	-	HSE06	^70^

Together, these challenges highlight that the realization of altermagnetism in coordination frameworks is not limited by conceptual feasibility, but by the integration of structural precision, symmetry fidelity, experimental accessibility and energetic robustness within a single material platform. Importantly, each of these challenges is already being actively addressed within the broader MOF community, suggesting that the remaining barriers are technical rather than fundamental. As a result, the emergence of experimentally verified AM frameworks should be viewed not as a distant prospect, but as a realistic next step enabled by continued advances in reticular chemistry, materials synthesis and characterization.

### New Horizons: Emerging Directions and Unexplored Territories

Among the emerging directions for altermagnetism in coordination frameworks, layer stacking represents one of the most immediately impactful and chemically accessible strategies. Notably, many of the magnetic MOFs discussed throughout this Perspective adopt intrinsically layered, vdW-bonded structures, where individual magnetic layers are weakly coupled along the stacking direction. This structural motif naturally enables stacking engineering as an additional degree of freedom. By controlling stacking sequence or symmetry relations between adjacent layers, it becomes possible to generate or modify the spin space group operations required for altermagnetism without altering the in-plane lattice. Exploiting the layered nature of magnetic MOFs in this way provides a powerful and largely unexplored route to realize AM phases through interlayer symmetry design rather than purely 2D lattice engineering.

Atomic or molecular intercalation provides a closely related and experimentally validated route to manipulate altermagnetism in coordination frameworks.^101^ Importantly, several experimental studies have already demonstrated that the intercalation of metallic atoms or molecular species into layered MOFs can induce dramatic changes in both magnetic and electronic properties, including enhanced magnetic ordering temperatures, modified exchange interactions and substantial increases in electrical conductivity.^42,43,102^ These results establish intercalation as a powerful postsynthetic strategy to tune framework properties without altering the underlying lattice topology. Notably, in layered vdW inorganic materials, atomic intercalation has recently been shown to induce or stabilize AM phases by modifying interlayer symmetry relations and electronic filling. Intercalation of interfacial Co atoms in the layered structure of NbSe_2_ has shown to introduce AM spin splitting confirmed experimentally via spin-polarized ARPES in hybrid CoNb_4_Se_8_.^103^ This precedent strongly suggests that analogous strategies in layered MOFs could be exploited to engineer altermagnetism through controlled interlayer coupling and symmetry manipulation. Building on these experimental foundations, intercalation emerges not as a speculative concept, but as a realistic and versatile tool for tailoring symmetry-driven magnetic behavior in coordination frameworks.

Beyond chemical design, external stimuli offer a powerful and largely untapped route to manipulate magnetic order in MOFs. In particular, hydrostatic pressure has already been demonstrated to dramatically tune magnetic interactions in layered magnetic materials by modifying interlayer spacing, orbital overlap and charge redistribution without altering the underlying lattice symmetry.^104^ Recent experiments show that pressure can strongly enhance interlayer exchange, coercivity and magnetic robustness while preserving crystallographic symmetry and long-range order, establishing pressure as an effective control knob for magnetic energy scales in MOFs.^105–107^ Remarkably, Li_0.7_Cr­(pyz)_2_Cl_0.7_ layered magnetic MOF has shown pressure tuning with a coercivity coefficient up to 4 kOe/GPa, which represents a powerful platform for manipulating the magnetic properties of molecular frameworks.^108^ Closely related to pressure, mechanical strain provides an additional and highly promising handle. In inorganic materials, strain has already been shown to induce and control AM phases through symmetry lowering and band structure reconstruction.^109^ Interestingly, ReO_2_ conventional antiferromagnet has been predicted to possess a strain-induced AFM-to-AM transition, which is dominated by a prominent *d*-wave AM anisotropy.^110^ In coordination frameworks, strain is known to strongly affect spin state energetics and has been used to trigger spin crossover and spin transition phenomena,^111^ highlighting the pronounced sensitivity of MOF electronic structure to lattice deformation. By extension, pressure and strain driven modulation of interlayer coupling and orbital hybridization represent highly promising strategies to access or stabilize AM phases, particularly in vdW stacked frameworks where symmetry protected spin splitting is sensitive to subtle structural changes. Importantly, these approaches parallel well-established stimulus controlled magnetic phase engineering in inorganic layered magnets, suggesting clear and experimentally realistic pathways to explore altermagnetism beyond purely chemical tuning.

An additional and largely unexplored direction concerns the realization of metallic AM MOFs. To date, all reported AM framework proposals correspond to semiconducting systems, in which the Fermi level lies within the band gap. This electronic structure requires external tuning, such as electrostatic gating or chemical doping, to shift the Fermi level toward the valence or conduction bands in order to experimentally access spin dependent transport or momentum resolved signatures.^20^ In contrast, intrinsically metallic AM frameworks would host symmetry driven spin splitting directly at the Fermi surface, enabling straightforward electrical detection and device integration. The development of metallic AM MOFs therefore represents a major opportunity for the field, combining the symmetry design principles of coordination chemistry with the transport functionality required for practical spintronic applications.

Heterostructure engineering represents another highly promising route for realizing and controlling altermagnetism in low-dimensional magnetic materials.^112,113^ In inorganic layered materials, the creation of vdW heterostructures has already been shown to induce AM states through interfacial symmetry breaking, band rearrangement and proximity effects, enabling electric-field control and ferroic switching of AM spin splitting.^114–116^ These advances provide a compelling blueprint for MOF-based systems, where substrate coupling has been predicted to drive magnetic phase transition due to charge transfer and strain.^117^ Importantly, experimental heterostructures combining 2D MOFs with inorganic 2D materials have already been demonstrated, establishing the feasibility of integrating molecular magnets into layered hybrid architectures. For example, a monolayer of magnetic Cu-dicyanoanthracene has been deposited in the surface of the superconductor NbSe_2_, which has shown to exhibit different structural arrangements which could be further coupled with magnetic and superconducting properties.^118^ In such systems, interfacial charge transfer, electrostatic coupling and symmetry lowering at the heterointerface offer powerful handles to activate or modulate altermagnetism without altering the intrinsic framework chemistry. This positions MOF-based heterostructures as a natural extension of current AM design strategies, combining molecular-level tunability with interfacial band and symmetry engineering.

Twist engineering has become a well-established strategy in inorganic vdW materials, where relative rotational misalignment between layers provides a powerful handle to modify symmetry, electronic structure and collective quantum states.^119^ In contrast, this approach has remained essentially unexplored in MOFs, despite the fact that many layered MOFs are held together by weak vdW interactions and are therefore, in principle, amenable to similar manipulations. Recently, the first experimental demonstration of twist engineering in 2D MOFs has been reported, showing that mechanically exfoliated MOF layers can be deterministically stacked with controlled relative orientation, leading to pronounced modifications of anisotropic physical properties.^120^ Although demonstrated so far in the context of optical anisotropy, this work establishes the experimental feasibility of twist-controlled MOF heterostructures. Extending this rotational control to magnetic frameworks naturally bridges the gap toward nonperiodic symmetry regimes. Indeed, recent works have highlighted alternative routes to altermagnetism beyond conventional crystalline frameworks, including quasicrystalline systems with noncrystallographic rotational symmetries and even amorphous or noncrystalline lattices where AM order emerges from local spin–orbital symmetry breaking rather than global lattice symmetry.^121–123^ Building on these concepts, MOFs emerge as a promising platform to explore such unconventional mechanisms. Thanks to their remarkable structural flexibility and chemical tunability, coordination frameworks can be intentionally designed as quasiperiodic Moiré architectures^124^ or geometrically frustrated amorphous networks.^125^ In these systems, global translational symmetry is structurally bypassed while highly controlled local coordination environments are preserved. These features create a promising framework to investigate AM phases beyond conventional crystallographic paradigms.

Finally, coordination frameworks offer a unique opportunity to explore altermagnetism in quasi-1D architectures. Recent theoretical work has demonstrated that AM states can arise in assemblies of parallel magnetic chains, where the relative arrangement, interchain coupling and symmetry relations between chains play a decisive role.^126^ Crucially, many MOFs are already known to host highly anisotropic magnetic lattices, featuring well-defined quasi-1D chains that are weakly coupled through organic linkers.^120,127^ These materials naturally realize the key ingredients identified in 1D AM proposals, namely spatially separated chains with tunable interchain spacing and magnetic coupling. As such, MOFs provide a chemically accessible and structurally versatile platform to embed 1D altermagnetism within an extended crystalline host, offering a promising bridge between recent theoretical predictions and experimentally realizable low-dimensional AM systems.

## Conclusions and Outlook

The emergence of altermagnetism opens a new frontier in magnetic materials design, where symmetry engineering becomes a central guiding principle. In this Perspective, we discuss that MOFs provide a uniquely powerful and timely platform to realize, generalize and ultimately control altermagnetism through chemical design. Unlike conventional inorganic magnets, coordination frameworks allow symmetry, lattice topology and electronic structure to be encoded at the molecular level, transforming symmetry from a fixed constraint into an actively tunable design parameter.

By surveying recent theoretical proposals and experimental advances, we have shown that the symmetry requirements for altermagnetism can be fulfilled across a broad range of framework architectures, encompassing 3D bulk frameworks, layered vdW-bonded materials, genuinely 2D networks and emergent quasi-1D motifs. More importantly, we have emphasized that the modular nature of MOFs enables systematic exploration of AM design principles beyond isolated model lattices, opening access to stacking engineering, intercalation, external stimuli, heterostructures and orbital-level control strategies that are largely inaccessible in dense inorganic solids. These opportunities position coordination frameworks as a platform to expand its phenomenology and functional scope.

The challenges described in this Perspective are not fundamental barriers, but technical and materials-oriented questions that are already being addressed within the rapidly evolving MOF community. The convergence of advances in reticular chemistry, electronic structure engineering and symmetry-sensitive characterization techniques suggests that the experimental realization and control of AM states in coordination frameworks is feasible. In this context, bridging symmetry-driven design with experimentally accessible synthetic strategies will be essential to translate theoretical proposals into realizable materials. We therefore anticipate that magnetic MOFs will play a central role in shaping the next phase of altermagnetism research, bridging symmetry-driven magnetic order with chemically programmable quantum materials and device-oriented functionalities.
